# Clinical Predictors of Testicular Torsion in Patients with Acute Scrotum; a Cross-Sectional Study

**DOI:** 10.22037/aaem.v10i1.1484

**Published:** 2022-01-11

**Authors:** Mohammad Sazgar, Seyed Hossein Montazer, Seyed Mohammad Hosseininejad, Fatemeh Jahanian, Behkam Rezaimehr, Mohammad Behbohaninia, Hamed Aminiahidashti

**Affiliations:** 1 Department of Emergency Medicine, Mazandaran University of Medical Sciences, Sari, Iran.; 2 Orthopedic Research Center, Mazandaran University of Medical Sciences, Sari, Iran.; 3 Diabetes Research Center, Mazandaran University of Medical Sciences, Sari, Iran.; 4 Department of Urology, Mazandaran University of Medical Sciences, Sari, Iran.; 5 Student research Committee, Faculty of Medicine, Mazandaran University of Medical Sciences, Sari, Iran.

**Keywords:** Scrotum, Acute Pain, Spermatic Cord Torsion, Early Diagnosis, Emergency Service, Hospital

## Abstract

**Introduction:**

Testicular torsion is an important and critical issue in patients with acute scrotum referring to emergency department (ED). Early detection is very important to save the testicles. This study aimed to determine the diagnostic accuracy of clinical variables in predicting the presence of testicular torsion.

**Methods:**

This prospective cross-sectional study was done using the information of patients hospitalized from September 2015 to September 2020, with complaint of acute scrotum (ICD 10 code: N50.8), referring to ED for evaluation of the clinical predictors of testicular torsions, which were confirmed by surgery.

**Results:**

81 patients with the mean age of 20.07 ± 9.64 (3- 45) years were studied. After surgical exploration, 70 patients (86.4%) had testicular torsion. Patients with torsion had lower age (p < 0.0001), lower time from symptom to ED visit (p < 0.0001), sudden onset pain (p = 0.003), left side pain (p < 0.0001), and lower white blood cell (WBC) count (p = 0.001). The frequency of dysuria (p = 0.032), diarrhea/vomiting (p = 0.005), and fever (p = 0.002) was significantly lower in patients with torsion. The cremasteric reflex was absent in 57 (81.4%) cases who suffered from testicular torsion (p = 0.001). Based on the results of binary logistic regression analysis, age (B = -0.175, SE = 0.45; p < 0.0001) was the sole independent predictor of testicular torsion. The highest area under the receiver operating characteristics (ROC) curve in predicting the presence of torsion belonged to lower age [91.0 (95%CI: 83.2 – 98.7)], pain in left testis [0.931 (95%CI: 0.828-0.987)], and lower WBC count [0.805 (95%CI: 0.684-0.926)], respectively.

**Conclusion:**

It seems that clinical variables are not accurate enough to be considered as the sole predictor of testicular torsion and they should be used with caution and in combination with other available screening tools like Doppler ultrasonography in this regard.

## 1. Introduction

Acute scrotum or testicular pain is one of the most important problems in the emergency department (ED). Acute scrotum is defined as a sudden onset scrotum pain with or without edema and tenderness, which is a true surgical emergency due to the possibility of testicular torsion (1, 2). There are a wide range of differential diagnoses for acute scrotum, and early detection of testicular torsion is essential (3). Epididymo-orchitis, torsion of the appendix testis, and inguinal herniation are some of the causes of presenting with acute scrotum symptoms (4). In some medical centers, all patients with acute scrotum are surgically explored to rule out testicular torsion (5, 6). There is clinical guidance in this regard, but it is not accepted worldwide (7). Color Doppler ultrasonography of the testis is used as a fast method with high sensitivity and specificity in the diagnosis of testicular torsion (8, 9). However, false-negative results are possible in the early stage, incomplete torsion, and intermittent torsion; therefore, clinical signs should be considered (10). Nevertheless, differentiating testicular torsion from other causes of the acute scrotum in the emergency department is crucial (11) and diagnostic exploration should be performed if the diagnosis still remains in doubt (12). Based on the above-mentioned points, this study aimed to determine the diagnostic accuracy of clinical variables in predicting the presence of testicular torsion. 

## 2. Methods

***2.1 Study design and setting***

This prospective cross-sectional study was done using the information of patients hospitalized from September 2015 to September 2020, with complaint of acute scrotum (ICD 10 code: N50.8), referring to the ED of Imam Khomeini Hospital, Sari, Iran, affiliated to Mazandaran University of Medical Sciences. The association between clinical characteristics and presence of testicular torsion (confirmed via surgery by urologist) was studied. This study was approved by the Ethics committee of Mazandaran University of Medical Sciences (IR.MAZUMS.REC.94-1313). The research team adhered to the ethical principles of the Helsinki Convention regarding clinical studies. 

***2.2 Participants***

 All patients referring to the ED with the complaint of acute scrotum, which was initially diagnosed as testicular torsion based on International Classification of Disease, version 10 (ICD10) code: N44) and underwent surgical exploration were included. Patients with incomplete information, those diagnosed with conditions other than testicular torsion, and also cases with underlying testicular diseases, such as a testicular tumor, cryptorchidism, and history of surgery were excluded from the study. Informed consent was obtained from all eligible patients or their legal representatives. The executor has adhered to the all the principles of the Helsinki Declaration.

***2.3 Data gathering***

Data regarding baseline characteristics, clinical examinations, history taking, and operating room reports of all patients was extracted from patients’ profiles and collected in a data collection form. Variables such as age, type of pain, time from beginning of pain to ED presentation, dysuria, hematuria, nausea and vomiting, side of pain, fever (T ≥ 38 ° C), cremasteric reflex, tenderness, erythema, and swelling were recorded for all cases. The data were collected by a trained emergency medicine resident under the direct supervision of an emergency medicine specialist. 

***2.4 Statistical analysis***

Considering sensitivity = 0.95 and specificity = 0.8 of redness and swelling of the scrotum and testicular pain (2, 13), 95% confidence interval, the maximum clinically acceptable width = 0.1, and prevalence = 0.25, the required sample size for this study was calculated to be 81 cases. Data were analyzed using SPSS version 21.0 (SPSS Inc, Chicago, IL, USA). Quantitative variables were expressed using mean ± standard deviation or frequency (%). The association between variables and presence of testicular torsion was studied using Chi-square or student t test. Binary logistic regression analysis was done on variables with significant association to determine the independent predictors of torsion. Area under the receiver operating characteristics (ROC) curve of each variable in predicting the presence of testicular torsion was calculated and reported with 95% confidence interval. P < 0.05 was considered as an acceptable cut-off for statistical significance. 

## 3. Results

***3.1 Baseline characteristics of studied patients***

**Figure 1:**
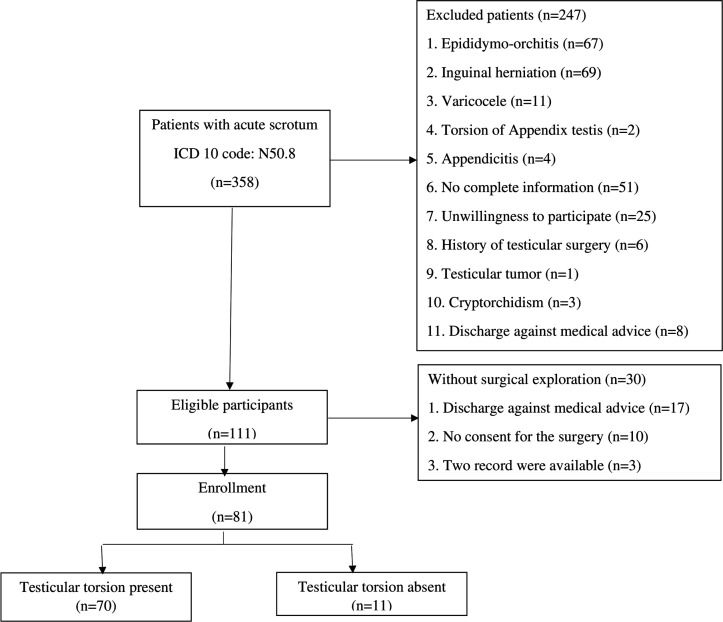
The flow chart of patients’ enrollment to the study

Out of the 358 patients referring to the ED with acute scrotum, 81 patients with the mean age of 20.07 ± 9.64 (range: 3- 45) years were suspected to have testicular torsion and were eligible for inclusion (Figure 1). The location of pain was the left testis in 67 (82.7%) cases, the right testis in 12 (14.8%), and on both sides in 2 (2.5%) patients. Testicular pain was sudden onset in 64 (79.01%) and gradual in 17 (20.98%) cases. Dysuria, vomiting, and fever was detected in 17 (20.98%), 35 (43.20%), and 21 (25.92%) cases, respectively. The mean time from the onset of symptoms to ED visit was 6.41 ± 11.01 (range: 1-72) hours. In clinical examinations, 20 (24.69%) patients had cremasteric reflex, 62 (76.54%) erythema, 68 (83.95%) testicular swelling, and 73 (90.12%) patients had testicular tenderness. Mean WBC count was 10700 ± 4400 cells/cubic millimeter. 20 (24.69%) patients underwent orchidectomy.

***3.2 Screening characteristics of clinical findings***

**Figure 2:**
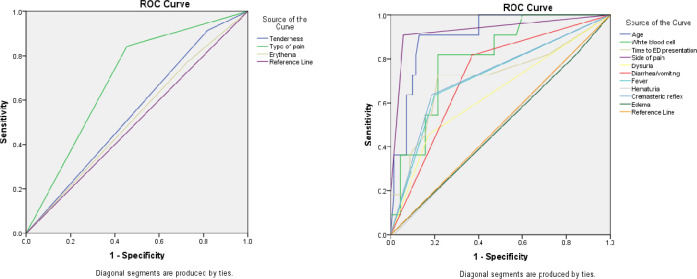
Area under the receiver operating characteristics (ROC) curve of different signs and symptoms in predicting the presence of testicular torsion (the values with 95% confidence interval are presented in table 2)

After surgical exploration, 70 patients (86.4%) had testicular torsion. Table 1 compares the baseline characteristics as well as signs and symptoms between cases with and without testicular torsion. Patients with testicular torsion had lower age (p < 0.0001), lower time from symptom to ED visit (p < 0.0001), sudden onset pain (p = 0.003), left side pain (p< 0.0001), and lower WBC count (p = 0.001). The frequency of dysuria (p = 0.032), diarrhea/vomiting (p = 0.005), and fever (p = 0.002) was significantly lower in patients with torsion. The cremasteric reflex was absent in 57 (81.4%) cases who suffered from testicular torsion (p = 0.001). Based on the results of binary logistic regression analysis, age (B = -0.175, SE = 0.45; p < 0.0001) was the sole independent factor in prediction of testicular torsion. Figure 2 and table 2 show the area under the ROC curve of studied variables in predicting testicular torsion. Accordingly, the highest area under the ROC curve value in this regard belonged to lower age [91.0 (95%CI: 83.2 – 98.7)], pain on the left side [0.931 (95%CI: 0.828-0.987)], and lower WBC count [0.805 (95%CI: 0.684-0.926)], respectively.

## 4. Discussion

Based on the findings of present study, patients with testicular torsion frequently presented with sudden onset pain in left testicle. The frequency of dysuria, diarrhea/vomiting, and fever, as well as WBC count was significantly lower in these cases. Age was the sole independent predictive factor of testicular torsion in this series. The maximum accuracy of studied variables in predicting torsion belonged to lower age, left side pain, and lower WBC count. 

Numerous studies have shown that pain, tenderness, and positive color Doppler ultrasound had the highest sensitivity in diagnosis of testicular torsion. It has been shown that some clinical variables such as testicular swelling and stiffness, the lack of cremasteric reflex, and moving up of the testicle had had a combined negative predictive value of 100% (specificity 81%, sensitivity 76%) in predicting testicular torsion (8). Age and involvement of the left testicle were independent factors in diagnosis of testicular torsion in Fabian et al. study (9), but in this study, age was the sole independent predictor of torsion. 

It has been shown that irreversible damage to the testis occurs less frequently in patients with torsion referring to the ED within the first 6 hours of symptom onset and complete infarctions would occur in 90% of cases that present to ED after 24 hours (13). In this study, lower time to ED visit was significantly associated with the presence of testicular torsion.

Neutrophil to lymphocyte ratio (NLR) was found to have a sensitivity of 84% and specificity of 92% in predicting testicular torsion (10). Although an increase in white blood cell count has been shown to be an inflammatory marker in patients with testicular torsion (5), it cannot be used as a marker for differentiating various causes of acute scrotum syndrome (14). Also, in this study, patients with testicular torsion had a significantly lower WBC count. 

It could be concluded that relying on clinical variables as the sole indicator of torsion and need for exploration surgery is not acceptable and decision in this regard should be made with caution and in combination with other findings.

**Table 1:** Comparison of baseline characteristics and clinical findings between cases with and without testicular torsion

**Variables**	**Testicular torsion**		**P**
	**Present (n = 70)**	**Absent (n = 11)**	
**Age (year)**	17.92 ± 7.85	33.72 ± 8.94	<0.0001
**Time to ED visit (hour)**	4.35 ± 4.72	19.45 ± 26.39	<0.0001
**WBC (cells/mm3)**	10039.28 ± 4278.19	14709.09 ± 3439.89	0.001
**Type of pain**			
Sudden	59 (84.3)	5 (45.5)	0.003
Gradual	11 (15.7)	6 (54.5)	
**Signs and symptoms**			
Dysuria	12 (17.1)	5 (45.5)	0.032
Diarrhea/vomiting	26 (37.1)	9 (81.8)	0.005
Fever	14 (20.0)	7 (63.6)	0.002
Hematuria	1 (1.4)	0 (0.0)	0.690
Erythema	54 (77.1)	8 (72.7)	0.748
Tenderness	64 (91.4)	9 (81.8)	0.321
Edema	54 (84.3)	8 (81.8)	0.748
**Side of pain**			
Left	66 (94.3)	1 (9.1)	<0.0001
Right	4 (5.7)	8 (72.7)	
Both	0 (0.0)	2 (18.2)	
**Cremasteric reflex**			
Yes	13 (18.6)	7 (63.6)	0.001
No	57 (81.4)	4 (36.4)	

ED: emergency department; WBC: white blood cell count.

**Table 2:** Area under the receiver operating characteristics (ROC) curve of different signs and symptoms in predicting the presence of testicular torsion

**Test Result Variable(s)**	**Area**	**95% CI **		**SE**	**P value**
		**Lower **	**Upper **		
**Age**	0.910	0.832	0.987	0.040	<0.0001
**Side of pain**	0.931	0.828	1.000	0.053	< 0.0001
**White blood cell**	0.805	0.684	0.926	0.062	0.001
**Cremasteric reflex**	0.725	0.549	0.902	0.090	0.017
**Diarrhea/vomiting**	0.723	0.571	0.875	0.078	0.018
**Fever**	0.718	0.542	0.895	0.090	0.021
**Time to ED presentation**	0.715	0.524	0.906	0.098	0.023
**Type of pain**	0.694	0.508	0.880	0.095	0.039
**Dysuria**	0.642	0.450	0.833	0.098	0.133
**Tenderness**	0.548	0.355	0.741	0.098	0.610
**Erythema**	0.522	0.335	0.709	0.096	0.815
**Hematuria**	0.493	0.310	0.676	0.093	0.940
**Edema**	0.488	0.301	0.674	0.095	0.896

CI: Confidence interval; SE: standard error; ED: emergency department.

## 5. Limitations

The limitations of the study were the high number of patients who did not consent to surgery after being a candidate for surgical exploration and those who were discharged from the emergency room against medical advice. Also, since the studied center did not have a pediatric ward, most of the patients were adults. 

## 6. Conclusion

It seems that, clinical variables are not accurate enough to be considered as the sole predictor of testicular torsion and they should be used with caution and in combination with other available screening tools like Doppler ultrasonography in this regard.

## 7. Declarations

### 7.1 Acknowledgment

The authors’ thanks emergency department staff for their assistance in conducting the study.

### 7.2 Authors’ contributions

HA, SMM, BR contributed to the project development and study design. MB and HA contributed data collection and interpretation of the data. HA, MS, FJ contributed to data analysis. HA, MS, SMH developed the manuscript. HA, MS, SMH, BR critically edited and revised the manuscript. All authors read and approved the final manuscript.

### 7.3 Conflict of interest

There are no conflicts of interest.

### 7.4 Funding and supports

This study was extracted from a thesis project, which was financially supported by a grant (No: 1313) from Mazandaran University of Medical Sciences.
